# Effect of homogenization and solution treatments time on the elevated-temperature mechanical behavior of Inconel 718 fabricated by laser powder bed fusion

**DOI:** 10.1038/s41598-021-81618-5

**Published:** 2021-01-21

**Authors:** Eslam M. Fayed, Mohammad Saadati, Davood Shahriari, Vladimir Brailovski, Mohammad Jahazi, Mamoun Medraj

**Affiliations:** 1 Department of Mechanical, Industrial and Aerospace Engineering, Concordia University, 1515 Sainte-Catherine Street West, Montreal, QC H3G 2W1 Canada; 2 Department of Mechanical Engineering, École de technologie supérieure, 1100 Notre-Dame Street West, Montreal, QC H3C 1K3 Canada

**Keywords:** Materials science, Mechanical properties, Metals and alloys

## Abstract

In the present study, the effect of homogenization and solution treatment times on the elevated-temperature (650 °C) mechanical properties and the fracture mechanisms of Inconel 718 (IN718) superalloy fabricated by laser powder bed fusion (LPBF) was investigated. Homogenization times between 1 and 7 h at 1080 °C were used, while solution treatments at 980 °C were performed in the range from 15 to 60 min. The as-printed condition showed the lowest strength but the highest elongation to failure at 650 °C, compared to the heat-treated conditions. After heat treatments, the strength of the IN718 alloy increased by 20.3–31% in relation to the as-printed condition, depending on the treatment time, whereas the ductility decreased significantly, by 67.4–80%. Among the heat treatment conditions, the 1 h homogenized conditions at 1080 °C (HSA1 and HSA2) exhibited the highest strength and ductility due to the combined effects of the precipitation hardening and sub-structural changes. Further increases in the homogenization time to 4 and 7 h led to a decrease in the strength and significant ductility loss of the LPBF IN718 due to the considerable annihilation of the dislocation tangles and a greater precipitation of coarse MC carbide particles. Furthermore, it was found that the solution treatment duration had a crucial influence on the mechanical properties at 650 °C due to the increase in the grain boundary strength through the pinning effect of the intergranular δ-phase. In addition, the fracture mechanism of the LPBF IN718 was found to be dependent on the heat treatment time. Finally, this investigation provides a map that summarizes the effect of homogenization and solution treatment times on the high-temperature mechanical behavior of LPBF IN718 by relating it to the corresponding microstructural evolution. This effort strives to assist in tailoring the mechanical properties of LPBF IN718 based on the design requirements for some specific applications.

## Introduction

In the past decades, elevated-temperature applications have become more extensive, and have come to include different fields, such as aerospace, petrochemical, power plant, energy, and nuclear reactor industries^1,2^. Demand for materials withstanding harsh in-service conditions has consequently received considerable attention in these applications. For instance, the gas temperature in some hot zones of high-performance aircraft engines may reach 1090 °C^1^. Different cooling techniques have accordingly been developed to reduce the actual metallic component’s temperature to a level at which superalloys can still be used. Inconel 718 (IN718) is one of the superalloys that are widely used in different elevated-temperature applications because of its ability to retain high mechanical strength at high temperatures up to 650 °C, and high resistance to surface deterioration, even in corrosive environments^3,4^. Nowadays, over 50% of the total weight of advanced aircraft engines components are fabricated from the IN718 superalloy^1^.

The mechanical properties of IN718 superalloy are highly related to its microstructure, which itself consists of multiple phases, such as γ-matrix, which is the primary phase, along with γ′ (Ni_3_ (Al,Ti)), γ″ (Ni_3_Nb), Laves phase (Ni,Fe,Cr)_2_(Nb,Mo,Ti), δ-phase (Ni_3_Nb) and carbides (MC, M_23_C_6_ and M_6_C)^5^. Adjustment of the sizes, contents and distributions of these phases is key to obtaining enhanced mechanical properties. In order to attain high mechanical properties that meet in-service requirements, IN718 is post-processed through the full heat treatment regime of: (1) homogenization, (2) solid solution and (3) precipitation hardening^6^. The homogenization and solid solution treatments are carried out at temperatures between 980 and 1200 °C to dissolve detrimental phases, such as Laves phases, and release age-hardening elements into the γ-matrix, in addition to precipitation of the intergranular δ-phase. The precipitation hardening treatment is performed in two consecutive steps to precipitate the strengthening phases (γ′ and γ″). The first step is realized at a temperature between 704 and 899 °C, and the second is carried out at a temperature between 593 and 704 °C^6^.

To date, IN718 components have either been cast, wrought or made by powder metallurgy^7^. Nevertheless, with fast improvements of the performance of turbine engines in the aerospace and energy industries, complicated cooling features are continually added to IN718 components to improve their capability to withstand significant temperature increases inside the advanced turbine engines. For instance, more internal cooling channels have been included in turbine blades. Such design and cooling features increase the fabrication cost and the geometrical complexity of components, and in some cases, are even impossible to incorporate in the manufacturing process using existing traditional methods. Furthermore, the high hardness and poor machinability of the IN718 superalloy introduce several challenges during the conventional manufacturing of IN718 components^2^. Thus, more and more attention is being paid to additive manufacturing (AM) techniques as alternative fabrication methods for IN718 parts, especially in the aerospace and energy industries.

IN718 superalloy is characterized by its high weldability^6^, and is highly promising as a candidate processed using AM. Among all AM techniques, the laser powder bed fusion (LPBF) process has gained considerable attention in various industrial sectors due to its outstanding potential versus conventional manufacturing routes. Compared to conventional methods, the LPBF process presents significant advantages, such as (1) the ability to fabricate complex-shaped components without geometrical limitations, (2) a low buy-to-fly ratio (as little as 2) versus conventional manufacturing methods, where this metric can reach 50, (3) production time and cost savings, and (4) the production of fine microstructures and improved mechanical properties as compared to traditional fabrication methods, such as casting and powder metallurgy^8–11^. Furthermore, the LPBF process is capable of processing various engineering metallic materials, including IN718, with promising mechanical properties. However, LPBF-fabricated IN718 components usually require thermal post-treatment to reduce their level of residual stresses, homogenize their microstructure and precipitate the desired phases to enhance their mechanical properties, especially at elevated temperatures. On the other hand, it has been found that applying improper heat treatment conditions can lead to the retention of some of detrimental phases, such as Laves phase, which could have a negative impact on the mechanical behavior of LPBF IN718^10^. Thus, suitable post-treatments are required to obtain a homogenized microstructure as well as high mechanical properties at elevated temperatures.

Although IN718 superalloy is mainly designed for elevated-temperature applications, the mechanical behavior of the LPBF IN718 reported in the literature is characterized mainly at room temperature^12–20^, and only a few works have been reported on its behavior at elevated temperatures^9,11,21,22^. For instance, Trosch et al.^11^ investigated the mechanical behavior of forged and LPBF-fabricated IN718 at room temperature (RT) and at 650 °C. Both were heat-treated using the standard heat treatment (SHT) conditions of wrought IN718 (solution annealing at 980 °C for 1 h + double aging at 720 °C for 8 h and at 620 °C for 8 h; designated as SA). Their results^11^ illustrate that at RT, the LPBF IN718 samples exhibited a higher tensile strength (TS), equivalent yield strength (YS), and lower ductility than did the wrought IN718. However, at 650 °C, the LPBF IN718 showed a significant drop in both strength and ductility as compared to the wrought alloy. The authors of this study^11^ attributed this behavior to the precipitation of intragranular δ-phase after the SHT of LPBF IN718. Thus, they suggested that homogenization treatment above 1030 °C (the solvus temperature of δ-phase), before the solution annealing, would diminish the amount of intragranular δ-phase, and consequently improve the mechanical properties at high temperatures^11^. Furthermore, Strößner et al.^9^ studied the effect of different heat treatments on the mechanical properties at 650 °C of IN718 alloy fabricated by LPBF. Before tensile testing, two AMS standard heat treatment conditions were applied to the as-printed IN718: (1) AMS 5662 (solution treatment at 980 °C for 1 h, followed by air cooling, and then aging at 760 °C for 10 h, furnace cooling to 650 °C with a 50 °C/h heating rate, and holding at 650 °C for 8 h with subsequent air cooling to RT); (2) AMS 5664 (homogenization treatment at 1065 °C for 1 h, followed by the same cycle of the solution and aging treatments as described in AMS 5662). Their results revealed that at 650 °C, the heat-treated LPBF IN718 exhibited a tensile strength (1115 MPa) comparable to that of its wrought equivalent, but a significantly lower elongation to failure (8%)^9^. Such elevated-temperature ductility loss (embrittlement) of the nickel-based superalloy fabricated by LPBF has been extensively reported in the literature^9,11,22,23^. In this context, Zhao et al.^22^ investigated the evolution of the tensile properties of the conventionally heat-treated (SA) LPBF IN718 at different temperatures (500, 550, 600 and 650 °C) to demonstrate the cause of the elevated-temperature ductility loss. Their^22^ results show that 650 °C is the critical temperature of the embrittlement phenomenon of the LPFB fabricated IN718 due to the excessive grain boundary oxidation and the overaging behavior at this temperature.

The application of the industrial standard treatments developed for either cast or wrought IN718 to their LPBF equivalent appears to be inadequate to improve the high-temperature mechanical properties of the latter. This is attributed to dramatic differences in the initial microstructure obtained after the LPBF, casting and forging manufacturing methods. In our previous study^24^, a post-heat treatment time window covering a wide range of soaking times for the homogenization (from 1 to 7 h at 1080 °C) and solution (from 15 to 60 min at 980 °C) treatments was established, and the effects of these post-treatment conditions on the microstructure, material texture, precipitates and hardness were studied. The objective of that study^24^ was to tune the homogenization and solution treatment times to improve the microstructure of LPBF IN718 components, with the ultimate goal of optimizing their mechanical properties. It was found that after the homogenization treatment for 1 h at 1080 °C, Laves phases and elemental segregation formed during the printing process were not completely dissolved, and the as-printed texture and grain structure were not significantly affected^24^. However, an increase in the homogenization treatment time at 1080 °C significantly influenced the microstructure in terms of precipitates and texture^24^, and the longer the duration of this treatment, the more intensive the dissolution of Laves phase, the diffusion of segregates and texture weakening^24^. A completely recrystallized material and stress-relieved equiaxed grains were obtained after 4 h of homogenization treatment at 1080 °C^24^. From a negative perspective, more and coarser carbides were observed after the 4 and 7 h homogenization times at 1080 °C^24^. Furthermore, with a 1 h homogenization treatment at 1080 °C, it was found that the solution treatment time significantly affected the fraction of δ-phase precipitation along the grain boundaries, whereas at the prolonged homogenization time (7 h), the significance of the solution time decreased^24^. However, the effect of the solution treatment time on the material hardness at RT was not altogether significant in both cases^24^.

As discussed above, changes in the homogenization and solution treatment times had a significant impact on the microstructural characteristics of the LPBF IN718, which consequently affected the mechanical properties. However, RT hardness testing did not provide comprehensive information about the effect of the evolution of the phases under different homogenization and solution times on the mechanical behavior of the LPBF IN718^24^. For instance, the effect of δ-phase and the precipitation of carbides on the material strength and ductility, especially at high temperature, was not captured in our previous study^24^. Moreover, only limited studies have been published on the elevated-temperature mechanical behavior of the additively manufactured IN718 superalloy, hence the need for further studies to optimize the homogenization and solution treatment times.

The present study is a logical continuation of our previous work as we assess the elevated-temperature mechanical properties and the fracture mechanism of the LPBF IN718 as a function of the homogenization and solution treatment times. This will allow a correlation with, and further understanding of the effect of the microstructure and phase evolution obtained in the former study on the in-service mechanical behavior.

## Material and methods

**Figure 1:**
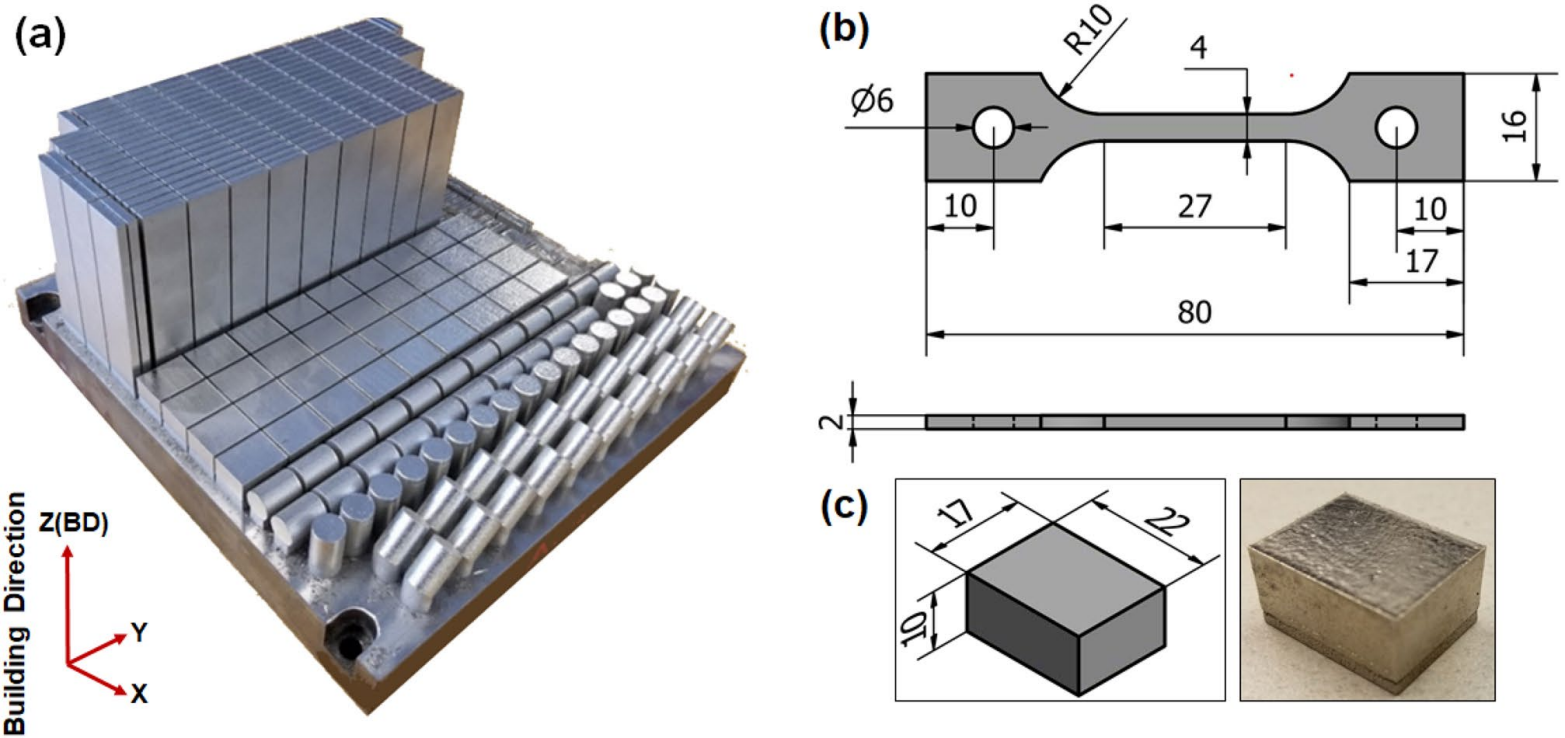
(**a**) Building platform illustrating the building orientation of the prismatic coupons; (**b**) geometry of the tensile samples; (**c**) geometry of the cuboid samples (dimensions in mm).

IN718 tensile samples were additively manufactured form a gas-atomized IN718 powder with a particle size distribution of D10 (18.2 μm), D50 (32.4 μm) and D90 (54.2 μm) using an EOS M280 (EOS, Krailling, Germany) LPBF system equipped with a Yb:YAG 400 W fiber laser. A nominal chemical composition of the as-received IN718 powder is as follows (wt. %): Ni (49.19), Cr (19.04), Nb (4.92), Mo (2.70), Al (0.33), Ti (1.08) and Fe (balance). The EOS Original Parameter Set IN718_Surface 1.0 (285 W laser with 100 µm beam diameter, 1000 mm/s scanning speed, 110 µm hatching space, and 40 µm layer thickness, and argon atmosphere) was used to manufacture two sets of coupons: 83 × 19 × 4 mm^3^ for tensile testing and 22 × 17 × 10 mm^3^ for microstructure analysis, as shown in Fig. 1. As can be seen in Fig. 1a, the coupons’ longitudinal axes were oriented parallel to the building direction. Wire electro-discharge machining (WEDM) was used to remove the coupons from the printing platform, and no stress relief heat-treatment was applied to preserve the as-printed microstructure.

**Figure 2:**
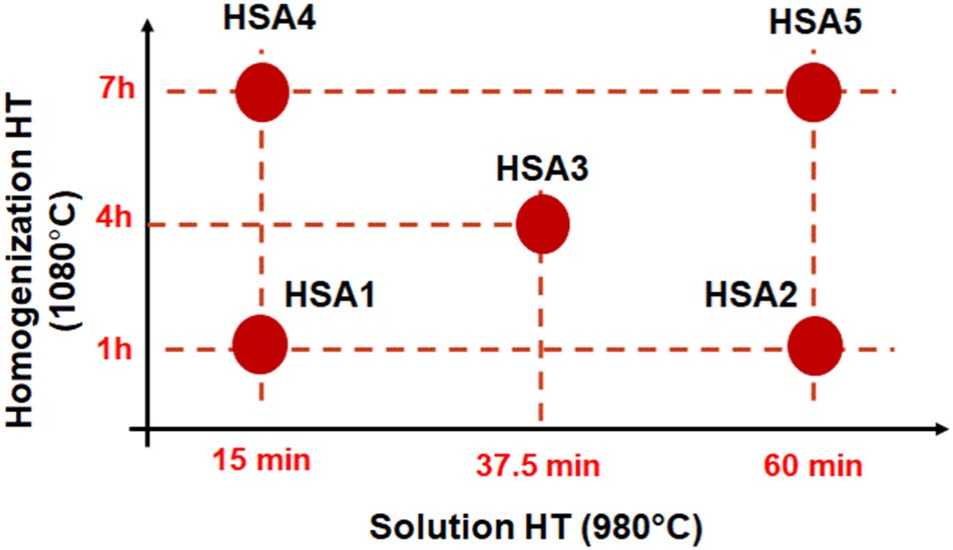
Schematic drawing shows the time window of post heat treatment conducted in the present study.

To investigate the effect of both homogenization and solution treatment times on the high-temperature tensile properties of LPBF IN718, a treatment time window, consisting of five conditions, was established, as shown in Fig. 2. This window combines a homogenization treatment time range at 1080 °C from 1 to 7 h, whereas for the solution treatment at 980 °C, it goes from 15 to 60 min. Therefore, as can be seen in Fig. 2, two samples were homogenization heat-treated at 1080 °C for 1 h: one of them was followed by solution treatment at 980 °C for 15 min (HSA1), and the other, for 60 min (HSA2), at the same temperature (980 °C). Similarly, the other two samples were homogenization heat-treated at 1080 °C for 7 h: one of them was followed by solution treatment at 980 °C for 15 min (HSA4) and the other, at 60 min (HSA5). Furthermore, a heat treatment condition with an intermediate time for homogenization and solution treatments was performed at 4 h for homogenization and 37.5 min for solution treatment (HSA3) to follow the evolution of the mechanical properties within the time window. Then, the corresponding samples were aged at 720 °C for 8 h, followed by furnace cooling at 55 °C/h to 620 °C, and then held at 620 °C for 8 h, followed by air cooling to room temperature. The designations and details of the five heat treatment conditions are listed in Table 1. Further justifications for these heat treatment conditions are given in our previous study^24^. The IN718 coupons were heat-treated with an electric-resistance furnace under normal atmosphere using a set of K-type thermocouples to monitor the coupons’ temperature during the treatment, with the temperature difference controlled within ± 5 °C. The coupons were finally WEDM-machined to the final dimensions shown in Fig. 1b. It is worth mentioning that the machining was realized after the heat treatments to avoid sample distortion due to the presence of residual stresses inherited from the printing process.

**Table 1:** Designations of the specimens and the details of the corresponding post-treatment conditions.

Designation	Homogenization heat treatment (H)	Solution heat treatment (S)	Aging heat treatment (A)
As-printed	None	None	None
HSA1	1080 °C for 1 h/AC	980 °C for 15 min/AC	720 °C/8 h/FC at 55 °C/h to 620 °C + 620 °C/8 h/AC
HSA2	1080 °C for 1 h/AC	980 °C for 1 h/AC	
HSA3	1080 °C for 4 h/AC	980 °C for 37.5 min/AC	
HSA4	1080 °C for 7 h/AC	980 °C for 15 min/AC	
HSA5	1080 °C for 7 h/AC	980 °C for 1 h/AC	

*AC* air cooling, *FC* furnace cooling.

**Figure 3:**
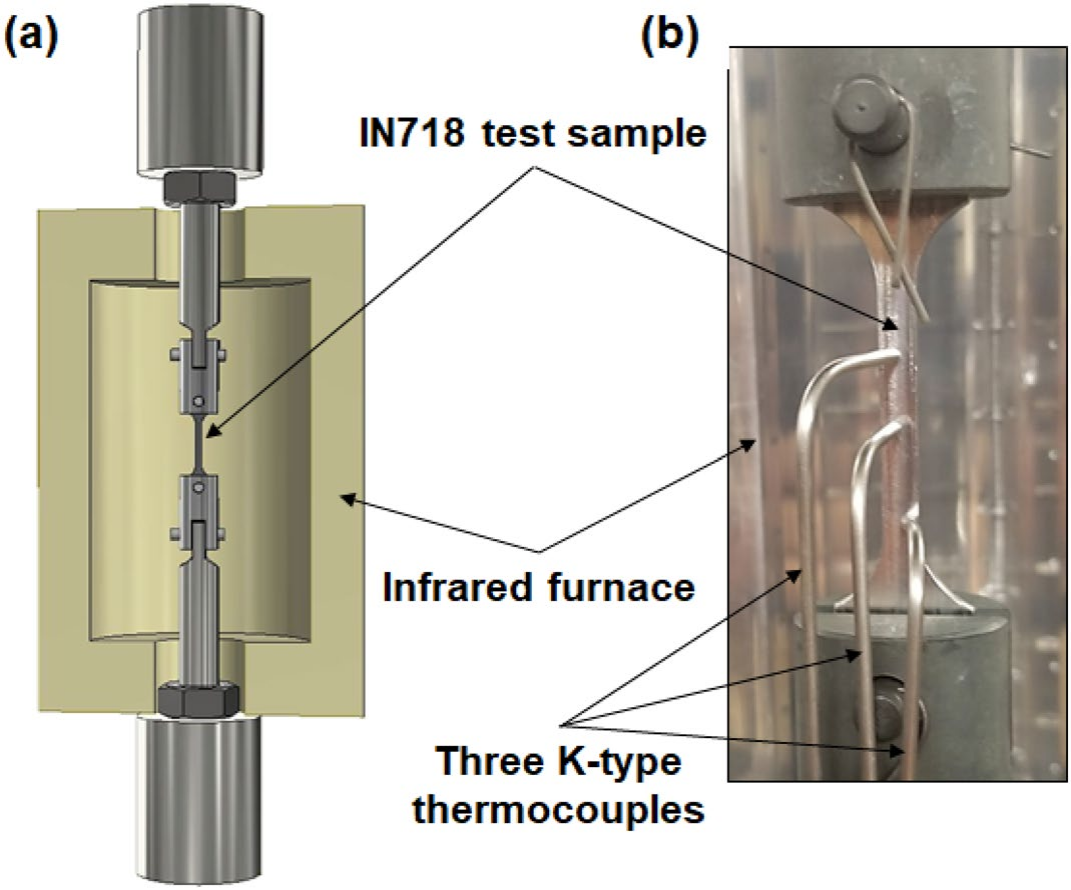
(**a**) Schematic illustration of the high-temperature (650 °C) tensile testing setup; (**b**) actual IN718 test sample with thermocouple arrangement.

Since the IN718 superalloy is mainly designed to fabricate components used in high-temperature applications, tensile testing at 650 °C was conducted to allow greater representation of the mechanical behavior of LPBF IN718 alloy in the in-service environment. To this end, an MTS 810 tensile testing system equipped with an infrared radiant heating furnace was used, as shown in Fig. 3. The tensile tests were conducted under a constant strain rate of 10^–3^ s^−1^. The IN718 tensile specimens were heated to 650 °C with a 1 °C/s heating rate, and then held for 10 min before testing to guarantee thermal uniformity along the total length of the specimen. All tested samples were pre-loaded with a constant force of 50 N before testing to prevent the samples from buckling as a result of the thermal expansion of the IN718 during heating to 650 °C. Three K-type thermocouples were used to monitor the thermal distribution along the gauge length. As can be seen in Fig. 3b, one of these thermocouples was placed in contact with the center of the gauge length, while the others were placed close to its upper and lower ends. The central thermocouple was used for temperature control during the tensile testing. For greater accuracy, two or three specimens were tested per condition. An argon atmosphere was applied with a flow rate of 4.7 L/min to minimize oxidation. After failure, a forced argon flow was provided immediately to cool the IN718 test samples to RT.

The tensile fracture and gauge length surfaces (surfaces perpendicular and parallel to the tensile loading direction, respectively) were analyzed using a scanning electron microscope (SEM), (HITACHI S-3400N) equipped with an energy dispersive X-ray spectroscopy (OXFORD, EDS) detector for the chemical composition analysis of primary and secondary phases. For the metallographic analysis of the gauge length surface, the tested samples, sectioned by a Buehler slow cutter, were mounted using a conductive hot epoxy resin. Then, the mounted samples were manually ground using SiC abrasive paper from 320 up to 1200 grit, followed by polishing down to 0.5 µm, using an alcohol-based diamond suspension. After polishing, a solution of 10 ml hydrochloric acid + 1.5 ml 30% hydrogen peroxide was used to etch the polished samples^25^. The evolution of the grain structure and misorientation angles with different heat treatments was examined by a SEM (SU-8230 HITACHI) equipped with a Bruker e^−^Flash HR^+^ electron backscatter diffraction (EBSD) detector using 20 kV and a pixel size of 1.62 μm. The total map size for each condition was 1298 × 973.4 μm^2^ surface area, to cover the maximum number of grains. The EBSD data were post-processed using QUANTAX ESPRIT software. For the EBSD analysis, the specimens were mechanically polished down to 0.5 μm, followed by a vibromet polishing for 24 h using a 0.05 grit size colloidal silica. Then, the residual fine scratches and deformed surfaces were eliminated using an IM4000Plus ion milling system under 6 kV accelerating voltage and 25 rpm rotation speed for 40 min. It is worth mentioning that only HSA2, HSA3 and HSA5 treatment conditions have been selected to illustrate the evolution of the grain boundaries through EBSD. This was based on the XRD and SEM analyses in our previous study^24^ where the homogenization treatment time was found to have more significant impact on the grain structure and boundaries than the solution treatment time. Hence, to understand the effect of homogenization time on the grain structure, HSA2, HSA3 and HSA5 treatments were selected representing 1, 4, 7 h homogenization time, respectively.

## Results and discussions

### The influence of the homogenization and solution times on the mechanical properties at 650 °C

**Figure 4:**
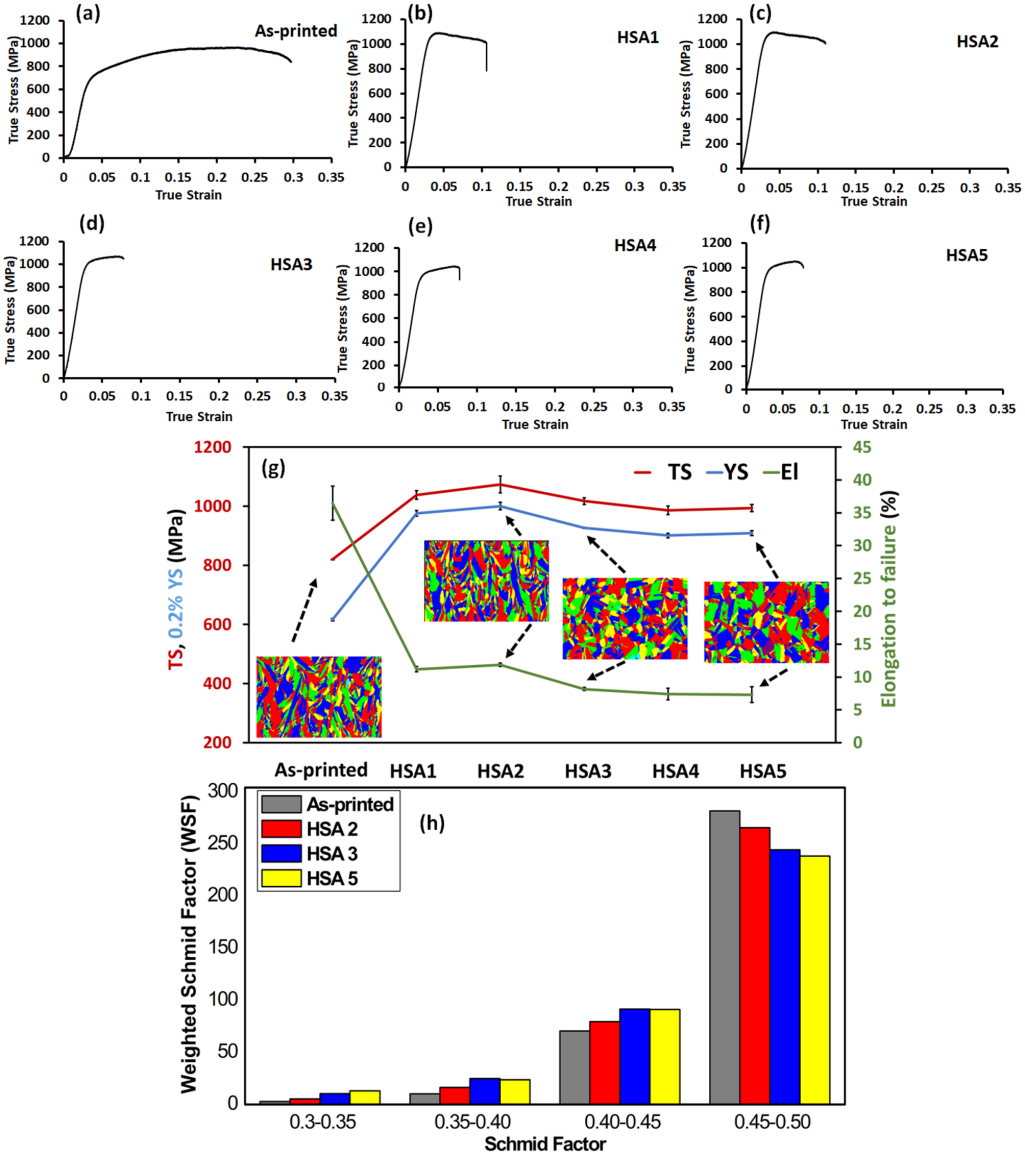
(**a**–**f**) True stress-true strain curves at 650 °C of the LPBF IN718 in the following conditions: (**a**) as-printed, (**b**) HSA1, (**c**) HSA2, (**d**) HSA3, (**e**) HSA4 and (**f**) HSA5; (**g**) evolution of the high temperature of tensile properties and the grain structure map as a function of the heat treatment conditions; (**h**) distribution of the Weighted Schmid Factor (WSF) under the as-printed and heat treatment conditions.

Figure 4a–g shows the high-temperature tensile (650 °C) properties of the LPBF IN718 in the as-printed and heat-treated conditions. The detailed average values of the mechanical properties are listed in Table 2. For comparison, the mechanical properties of the conventionally heat-treated (AMS) wrought, cast and LPBF-fabricated IN718 superalloy obtained at 650 °C by Gao et al.^26^, Trosch et al.^11^ and Zhao et al.^22^, respectively, are included in Table 2. As can be seen in Fig. 4a–f, the homogenization and solution treatment times affect the flow stress of the LPBF IN718 alloy significantly. A detailed explanation of the evolution of the flow stress is discussed in the next section.

**Table 2:** Elevated-temperature (650 °C) tensile strength (TS), yield strength (YS) and elongation to failure (El) values of the as-printed and heat-treated conditions.

Condition	TS (MPa)	YS (MPa)	El (%)	References
As-printed	820 ± 2	617 ± 3	36 ± 3	This work
HSA1	1038 ± 14	976 ± 9	11.2 ± 2	
HSA2	1074 ± 28	1001 ± 12	11.8 ± 0.3	
HSA3	1017 ± 11	927 ± 2	8.2 ± 0.2	
HSA4	986 ± 14	901 ± 7	7.4 ± 0.9	
HSA5	994 ± 11	909 ± 8	7.3 ± 1	
Heat-treated Wrought (AMS5662)	1000	862	12	^26^
Heat-treated Cast	576	517	13.7	^11^
LPBF IN718 AMS5662	1025	915	5.5	^22^
LPBF IN718 ASM5383	1020	950	3.5	^22^

The elevated-temperature (650 °C) tensile properties of the connectionally heat-treated (AMS) IN718 fabricated by LPBF, wrought and cast routes, reported in the literature^11,22,26^, are included for comparison.

Figure 4g illustrates the changes in the high-temperature mechanical properties and grain morphology of the LPBF IN718 as a function of the heat treatment conditions. It is worth mentioning that the grain maps included in this figure were acquired from the vertical plane (xz) with respect to the building direction (the plane parallel to the tensile loading direction). As can be seen in Fig. 4g, the as-printed condition exhibits the lowest tensile and yield strengths, as compared to those of the heat-treated LPBF IN718 in the present study and wrought (AMS5662) IN718 in^26^, while its elongation to failure is the highest. This can be attributed to a combined effect of a lack of strengthening phases (γ′ and γ′′) and the presence of Laves phase in the inter-dendritic regions, as well as at the grain boundaries, as reported in our previous study^24^. It is well-recognized that the precipitation of Laves phase in the microstructure degrades the high-temperature tensile properties. However, the tensile (820 MPa) and yield (617 MPa) strengths and the elongation to failure (36.4%) of the as-printed IN718; observed in the current work, are significantly higher than the tensile properties of the heat-treated cast IN718 at 650 °C (576 and 517 MPa and 13.7%), as reported by Trosch et al.^11^. This can be explained by the fine microstructure and microsegregation that result from the LPBF process of IN718 superalloy due to rapid solidification, as compared to the coarse microstructure and macrosegregation of the cast IN718, as reported by Zhang et al.^10^. The latter confirmed that the presence of such macrosegregation in cast IN718 is difficult to completely eliminate even after heat treatment, which consequently leads to the formation of larger irregular Laves phase in the inter-dendritic zones when compared to LPBF IN718^10^. Moreover, the precipitation of coarse δ-phase was observed after post-treatment of cast alloy, which also had an adverse impact on the mechanical properties^10^.

The high ductility of the as-printed condition, as compared to the heat-treated LPBF, wrought and cast IN718 equivalents, can also be attributed to the orientation of the elongated grains in the direction of tensile loading, as shown on the grains map in Fig. 4g [as earlier mentioned, the tensile samples were printed parallel to the building direction (Fig. 1a)]. Thus, there is less disruption of the motion of dislocations through the low angle grain boundaries during deformation, which consequently, results in higher elongation. In order to enlighten the influence of the favorable microstructure texture and the crystal orientation on the plastic flow and material elongation, the weighted Schmid factor (WSF), defined as the area under the Schmid factor curve in a given interval, was used. WSF represents the number of indexed pixels in an orientation imaging microscopy (OIM) analysis within a targeted Schmid factor range. It is worth noting that the zero-solution indexation for all studied EBSD maps were less than 0.3%, and therefore has a negligible impact on the WSF comparisons. As can be seen in Fig. 4h, the Schmid factor (SF) of the {111} <110> slip system has been considered in four intervals from 0.3 to 0.5. It can be observed that, at the highest SF interval (0.45 to 0.5), the as-printed condition exhibits a higher WSF as compared to the heat-treated conditions, indicating a lower resistance to deformation under loading along the z-direction (longitudinal axis of the tensile test coupons). This is attributable to the directional solidified microstructure and the strong texture along the building and the tensile loading directions (z-direction). Similar high as-printed elongations of the vertically-oriented LPBF IN718 are observed in^17,27,28^. It is worth mentioning that the calculated SF for the vertical as-printed samples reported in^17^ is approximately 0.47, which is consistent with that obtained in the present study.

Generally, any of the five heat treatments applied significantly increased the tensile and yield strengths, while ultimately decreasing the elongation to failure at 650 °C, as shown in Fig. 4g. For comparison, the 1 h homogenized treatments at 1080 °C in HSA1 and HSA2 increased the tensile strength by 26.5 and 31%, as compared to the as-printed conditions, whereas the elongation to failure decreased by 69 and 67%, respectively. It is important to note that, among all the heat treatment conditions, the HSA1 and HSA2 exhibited the highest tensile strength and elongation to failure. Also, as compared to the wrought IN718 (AMS5662), these conditions resulted in higher tensile and yield strengths and comparable elongations to failure. This is attributed to the greater precipitation with γ′ and γ′′ occurring after these conditions were applied than in the other heat treatments, as reported in our previous study^24^. However, precipitation hardening is not the only strengthening mechanism in the 1 h homogenized conditions. The presence of a high density dislocation and misorientation substructure also contributed to relatively higher mechanical properties when compared to other heat treatments.

**Figure 5:**
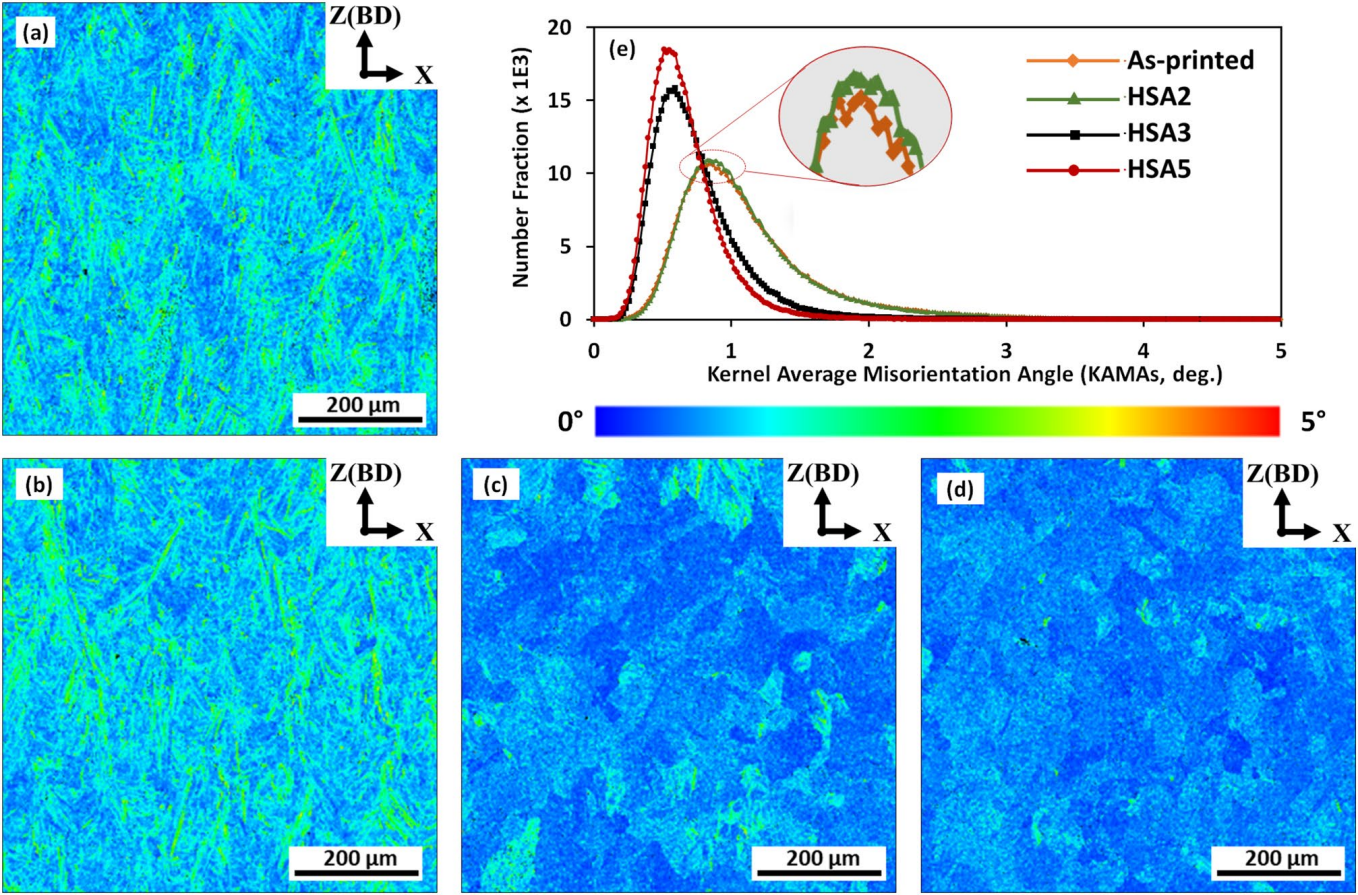
Kernel average misorientation (KAM) maps of the LPBF IN718 in the following conditions: (**a**) as-printed, (**b**) HSA2, (**c**) HSA3, (**d**) HSA5 and number fraction of KAM under as-printed and aforementioned heat treatment conditions.

Figure 5 shows the kernel average misorientation (KAM) maps of the LPBF IN718 in the as-printed and heat-treated conditions. KAM helps to estimate the plastic strain in individual grains and to reveal local variations in lattice orientations, and is a good indicator of the dislocation density in the material^29^. In the KAM maps in Fig. 5, the blue and red color codes represent the lowest and the highest misorientation angles/dislocation density, respectively. For quantification, the evolution of the number fraction (NF) of the KAM in the as-printed and the selected heat treatments is shown in Fig. 5e.

As can be seen in the KAM map in Fig. 5a, a high plastic strain intensity is induced in the initial microstructure of the as-printed condition, suggesting a high dislocation density resulting from the complex thermal cycling and rapid heating, followed by rapid cooling, during the printing process. Even after the 1 h homogenization condition, HSA2, is applied, a high intensity of the induced plastic strain is retained, as shown in Fig. 5b. Further, it can be observed from Fig. 5e that the as-printed and HSA2 exhibited nearly the same KAM values (≈ 0.83), higher than the heat-treated conditions (≈ 0.5). This clearly indicates that the 1 h homogenization treatment at 1080 °C is not sufficient to significantly annihilate the primary dislocation tangles inherited from the LPBF printing process, which consequently contributed to the high mechanical properties. After the HSA3 and HSA5 conditions, a significant reduction in the induced plastic strain is observed (Fig. 5c,d), indicating that the internal plastic strain is mostly relieved.

**Figure 6:**
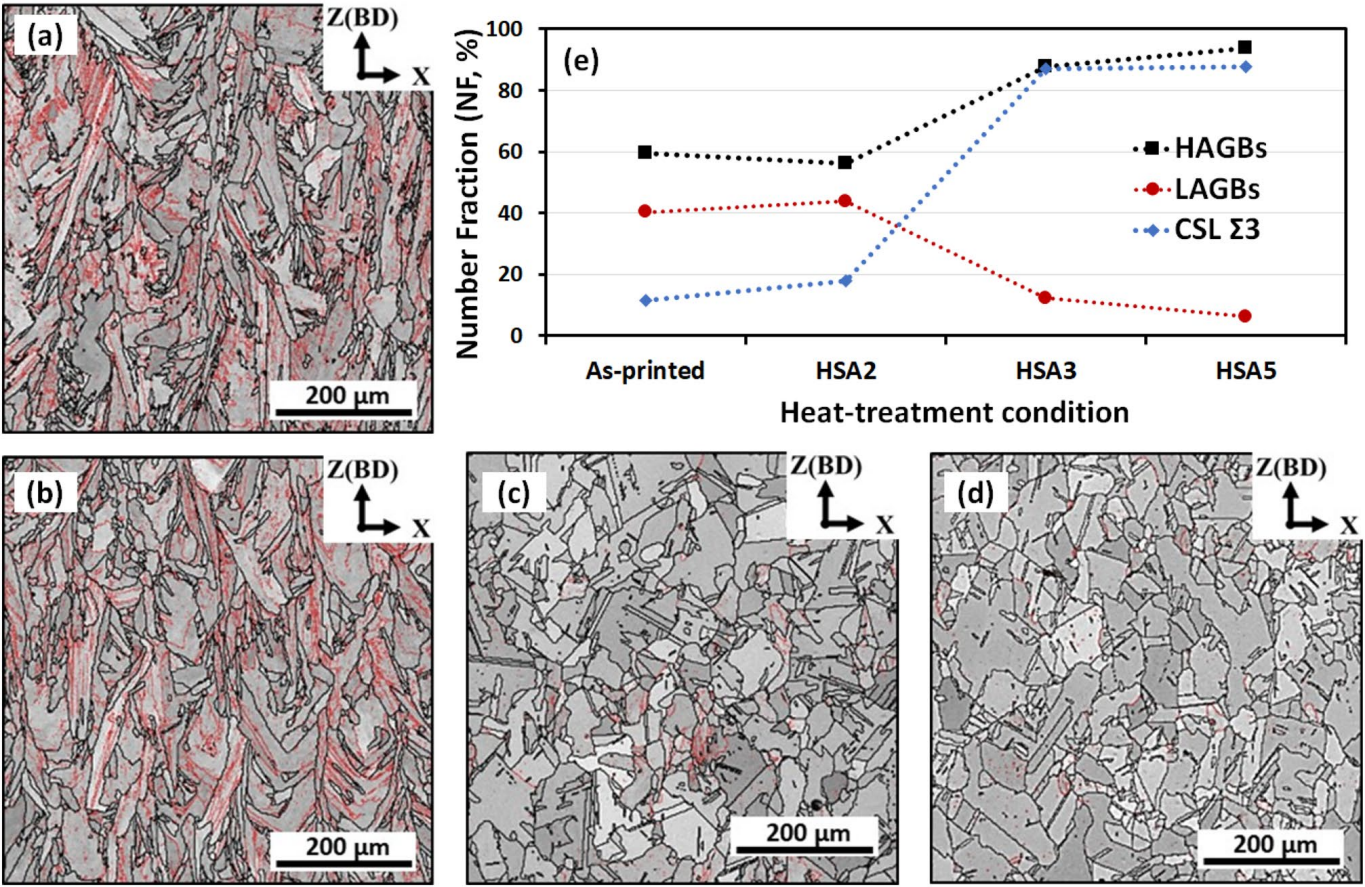
Distribution of LAGBs and HAGBs under different conditions: (**a**) as-printed, (**b**) HSA2, (**c**) HSA3, (**d**) HSA5, and (**e**) evolution of number fraction of LAGBs, HAGBs and CSL Ʃ3 grain boundaries under the aforementioned conditions. The LAGBs and HAGBs are indicated by red and black full lines, respectively.

Figure 6 also shows the evolution of the low and high angle grain boundaries, LAGBs and HAGBs, in the as-printed and heat treatment conditions, represented by red and black full lines, respectively. The misorientation angles (MAs) between 2° and 15° were defined as LAGBs, while the MAs greater than 15° were defined as HAGBs. As can be seen in Fig. 6a, the microstructure of the as-printed condition is composed of columnar grains with a relatively high density of LAGBs, when compared to the heat treatment conditions. This finding is consistent with the microstructure analysis by Fayed et al.^24^ of the as-printed condition, where it was found that elongated grains, composed of cellular and columnar dendrites, formed during the solidification process of LPBF, were divided by the LAGBs. After the 1 h homogenization treatment (HSA2), the columnar grains retained approximately the same LAGBs density as observed in Fig. 6b. Figure 6e shows the number fraction (NF) evolution of the HAGBs, LAGBs and the coincidence site lattice (CSL Ʃ3) grain boundaries in the as-printed and heat-treated conditions. It has been reported that the CSL Ʃ3 are special grain boundaries that are used to identify the twin boundaries and to study the grain boundary character distribution in EBSD analysis^30^. As can be seen in Fig. 6e, the as-printed and HSA2 conditions contain almost the same LAGBs density, with an NF of ≈ 40.4%. Furthermore, after the HSA2 treatment, a slight change in the CSL Ʃ3 grain boundaries is observed, indicating that the static recrystallization process only initiated but did not complete, which is consistent with the microstructure analysis reported in^24^.

When the homogenization treatment time at 1080 °C increases to 4 and 7 h, a significant decrease in LAGB density (Fig. 6c,d) is observed with NFs of 12.4% and 6.3%, respectively (Fig. 6e). Furthermore, a significant increase in the NFs of the CSL Ʃ3 and HAGBs is observed. It is interesting to note that the NFs of both the CSL Ʃ3 and HAGBs exhibited the same trends. However, a stable NF of the CSL Ʃ3 grain boundaries at ≈ 87.5% after the 4 h homogenization treatment is observed, indicating that complete recrystallization took place after the HSA3 condition. It can be concluded that the 4 h homogenization treatment at 1080 °C is the heat treatment condition necessary to trigger the complete recrystallization process and reduce or remove the existing internal stresses.

It is important to mention in this context that the high mechanical strengths of the HSA1 and HSA2 at 650 °C as compared to the other heat treatment conditions are consistent with the RT hardness results reported in^24^. Moreover, the relatively high elongations of the HSA1 and HSA2 conditions are attributable to the existence of elongated grains oriented parallel to the loading direction and the high SF as observed from the grains map and WSF distribution (Fig. 4g,h). The 1 h homogenization treatment at 1080 °C did not significantly change the as-printed texture and columnar grain structure, which contributed to the relatively high elongations measured for these conditions.

**Figure 7:**
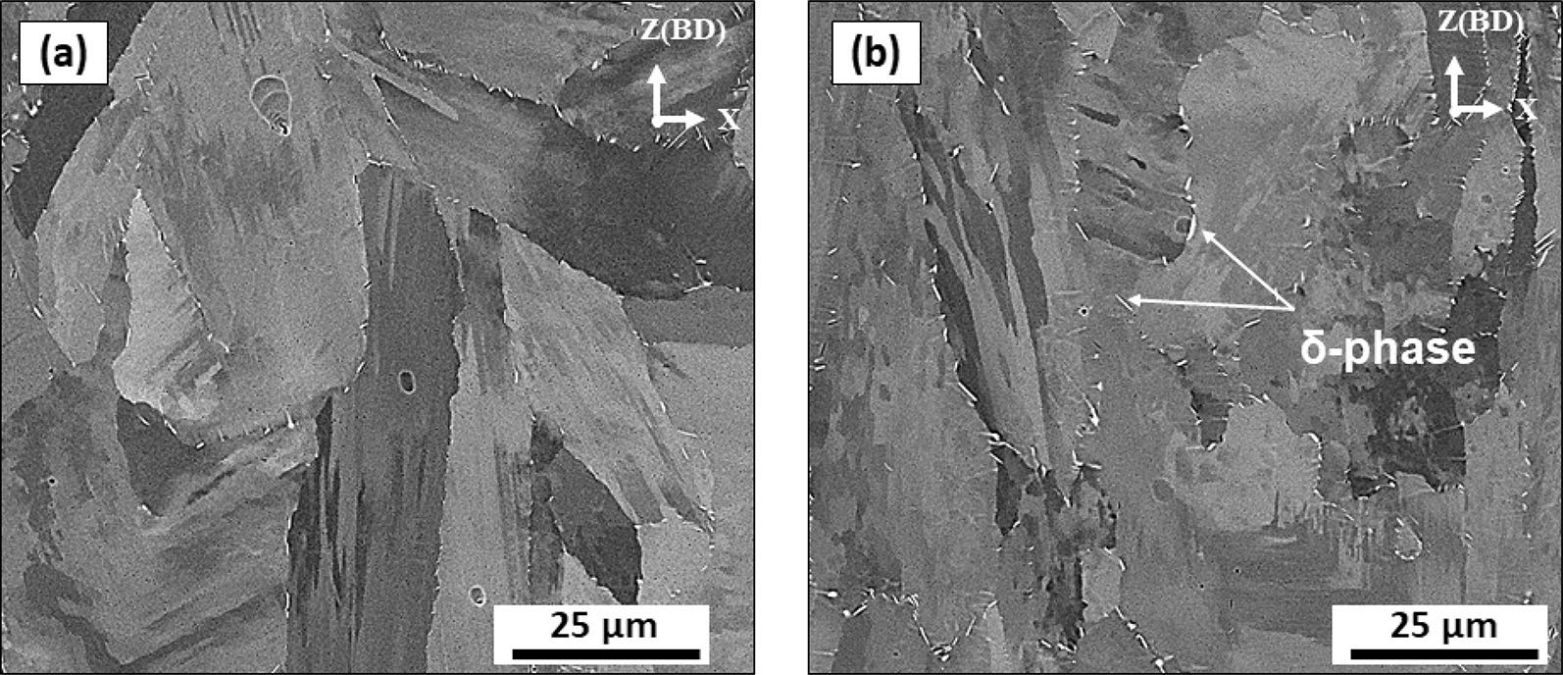
SEM micrograph of a non-etched LPBF IN718 illustrating the presence of δ-phase precipitated for heat treatment conditions: (**a**) HSA1; (**b**) HSA2.

On the other hand, after the 1 h homogenization treatments, it was found that the selected solution treatment times at 980 °C significantly affected the mechanical properties at 650 °C. For example, the HSA2 condition, which included a longer solution time (60 min), exhibited higher tensile and yield strengths than those of the HSA1 condition (15 min solution time). This behavior can be explained by higher δ-phase precipitation along the grain boundaries after HSA2, with an approximate area fraction of ≈ 0.66%, than after HSA1 (≈ 0.16%), as shown in Fig. 7. It is worth mentioning that the area fraction of δ-phase in those heat treatment conditions was quantified on the basis of digitized SEM micrographs and image threshold analysis using ImageJ software. Such a higher δ-phase fraction results in an increase in the grain boundaries’ strength through their pinning. During high-temperature deformation, δ-phase acts as a barrier to the dislocation motion, which consequently increases the tensile strength. Gao et al.^26^ also reported that a lack of δ-phase at the grain boundaries results in the high notch sensitivity seen at elevated temperatures. Nevertheless, having excess δ-phase is not favorable either, since it has a detrimental effect on the strength and ductility of the material, as reported in^31^. Thus, a moderate amount of δ-phase (≈ 4%)^32^ is required to ensure high mechanical strength characteristics at elevated temperatures.

**Figure 8:**
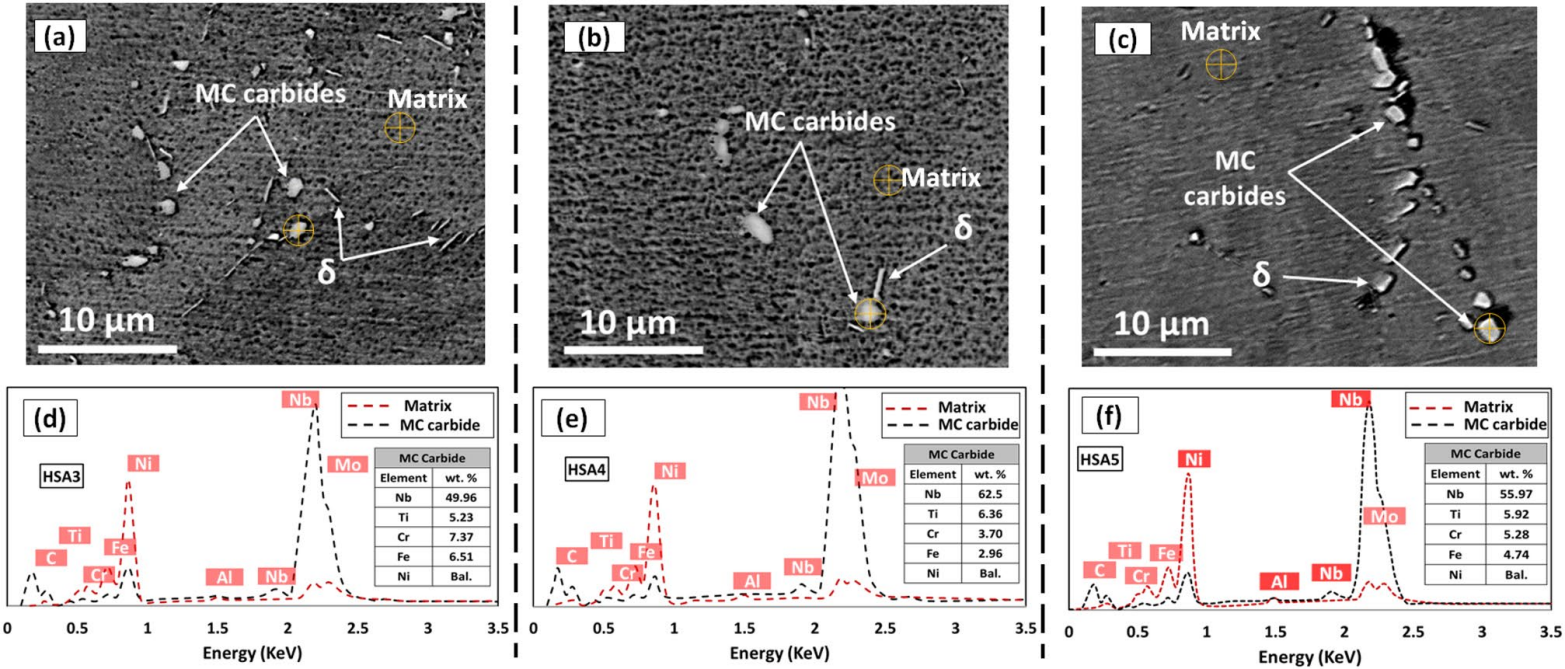
(**a**–**c**) SEM micrograph of LPBF IN718 in heat treatment conditions of: HSA3, HSA4 and HSA5, respectively, and (**d**–**f**) the corresponding EDS spectrum analysis diagram of the spots indicated in SEM micrographs, illustrating the concentration difference of the Nb between the γ-matrix and MC carbide.

When the homogenization treatment time increased to 4 h (HSA3), the strength of IN718 increased by 24% and became comparable with that of the wrought IN718 (AMS5662), whereas the ductility was significantly reduced, by 77.6%. However, the HSA3 treatment resulted in lower strength and ductility than those of both HSA1 and HSA2, as shown in Fig. 4g. This could be attributed to the combined effects of dislocation annihilation and the precipitation of coarse carbide particles, as shown in Fig. 8a. The KAM and misorientation maps in Figs. 5 and 6 clearly show the homogenization time increase to 4 h significantly decreased the induced plastic strain and grain substructure by triggering a complete recrystallization process after HSA3. Although a significant amount of γ′ and γ′′ forming elements were released through a further dissolution of Laves phase and the back diffusion of segregated elements after HSA3, a considerable amount of Nb and Ti was consumed in the precipitation of the carbide particles, resulting in the depletion of the γ-matrix, as shown in Fig. 8d. Therefore, a smaller amount of γ′′ and γ′ is expected to precipitate during the aging treatment in the HSA3 condition, as compared to the HSA1 and HSA2 conditions.

A further increase in the homogenization time to 7 h at 1080 °C in HSA4 and HAS5 conditions led to a significant drop in tensile and yield strengths and ductility, when compared to the other heat treatments and to the wrought IN718 (AMS5662). This is due to the combined effects of grain growth of the γ-matrix, the annihilation of dislocation density (Figs. 5d, 6d) and the coarsening of the carbide particles, as shown in Fig. 8b,c. Figure 8d–f shows the EDS analysis of the precipitated carbide particles after HSA3, HSA4 and HSA5 conditions. As seen in this figure, up to ≈ 60 wt. % of Nb and 6 wt. % of Ti are consumed in these carbide particles. Thus, the particles can be indexed as MC-type carbides according to the study by Mostafa et al.^33^ and Kreitcberg et al.^23^. It is worth mentioning that, among all carbide types in IN718 alloy, MC carbides form at high temperature, and are distinguished by their high Nb and Ti contents, unlike M_6_C and M_23_C_6_ carbides. Thus, the precipitation of MC carbides consumes a significant amount of Nb and Ti at the expense of the precipitation of γ′ and γ′′. More detailed discussions on the consistency of the lattice parameter variations of the matrix with the depletion of Nb and Ti in the γ-matrix of the HSA3, HSA4 and HAS5 heat treatments (EDS analysis) have been presented in^24^.

As can be seen, after the application of the HSA3, HSA4 and HSA5 conditions, the LPBF IN718 exhibited a significant ductility loss at 650 °C. This can be explained by the weakening of the grain boundaries’ strength, as many nickel-based superalloys often experience such embrittlement behavior at elevated temperature^23^. Several reasons have been advanced in the literature to explain the driving force for this phenomenon. These include precipitation of secondary carbides during hot deformation, micro-twinning during deformation, and interactions of dislocation and intermetallic particles^23^. Accordingly, all the aforementioned mechanisms are likely to be responsible for the embrittlement observed in the present study. However, further investigations are required to determine the dominant mechanism at play here.

### The effect of homogenization and solution treatment times on the flow stress at 650 °C

Figure 4a–f shows typical true stress–strain curves obtained at 650 °C for the as-printed and heat-treated conditions. The influence of the heat treatment holding time on the stress–strain curves can be clearly revealed seen. In Fig. 4a, the stress–strain curve of the as-printed condition exhibits three distinct stages, starting from the work hardening stage, in which a rapid increase in the stress with strain is observed, followed by a steady stress stage, in which the stress remains approximately constant, and finally, the softening stage which proceeds till failure.

Conversely, the flow stress of the 1 h homogenized conditions at 1080 °C (HSA1 and HSA2) shows a yield strength peak followed by work softening until failure, and the steady stress stage disappears completely, as shown in Fig. 4b,c. Such a rapid decrease in the mechanical strength can be explained by the occurrence of dynamic structural changes during the high-temperature deformation, and could be associated with dynamic recrystallization (DRX). For the IN718 superalloy, with its low to intermediate stacking fault energy^29,34,35^, the softening mechanism is mainly driven by the recrystallization process (i.e., with minor contribution from dynamic recovery). This is because, in metals with low stacking fault energy, the total dislocation network in the material decomposes into extended dislocations with large stacking fault widths, making their bundling very difficult during the recovery process^29^. Thus, dynamic recrystallization only becomes the dominant softening mechanism in IN718 once the critical dislocation density for recrystallization is attained. According to Fig. 5a,b, both the as-printed and 1 h homogenized conditions preserve a high density of the primary dislocation network inherited from the LPBF printing process. However, the as-printed conditions did not exhibit such DRX behavior, as seen in Fig. 4a. This finding indicates that the sum of the primary dislocation density and the accumulated dislocations generated during the hot deformation process were not enough to promote the DRX process, and that other mechanisms probably played a role in the occurrence of DRX. In the other words, the occurrence of the dynamic recrystallization process during the hot deformation of the HSA1 and HSA2 treated conditions could be attributed to another factor, combined with the primary and generated dislocation network.

**Figure 9:**
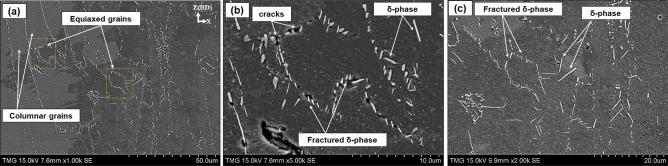
SEM micrograph displaying the longitudinal plane of the fracture surface of the HSA2 sample: (**a**) overall view of the fracture surface; (**b**,**c**) magnified view of the fracture surface as indicated in the overall view (**a**).

Kreitcberg et al.^23^ reported that the presence of a needle-like δ-phase along the grain boundaries during the hot deformation of nickel-based superalloys can accelerate dynamic recrystallization. Similarly, it has been reported by Wang et al.^36^ that the presence of the needle-like δ-phase in IN718 during plastic deformation increases the dislocation generation rate, which consequently promotes the dynamic recrystallization process. This is because of the pinning effect of the δ-phase at the grain boundaries, and the impediment of the dislocation movements during the plastic deformation, which induces strain gradients, and consequently initiates local recrystallization. The initial microstructure of both HSA1 and HSA2 conditions contains columnar grains with needle-like δ-phase along the grain boundaries^24^. The finely recrystallized equiaxed grains observed in the areas surrounded by δ-phase, as reported in Fig. 9, could therefore be explained by the above analysis.

Figure 9 shows the longitudinal cross-sectional plane of the fracture surface of HSA2 after tensile testing at 650 °C. As can be seen in Fig. 9a, the microstructure mainly consists of columnar grains oriented parallel to the loading direction, accompanied by needle-like δ-phase along the grain boundaries. Moreover, equiaxed grains with shorter δ-phase needles at the grain boundaries are similarly observed (Fig. 9b). This suggests that during the plastic deformation at 650 °C, the δ-phase needles impeded the dislocation movements and induced a gradient of plastic strain until reaching a critical point, after which new recrystallized equiaxed grains nucleated and formed as a result of the DRX process. The presence of such a short δ-phase (fractured δ-phase) along the new equiaxed grains could be attributed to the accumulation of the dislocations impeded by this phase and a high-level of stress concentration, which finally ended up forming cracks, leading to fracture. The above findings are in agreement with those of Hong et al.^37^, who also observed the presence of the fractured δ-phase along the boundaries of the new recrystallized grains.

Despite the needle-like δ-phase precipitated along the grain boundaries after the application of the HSA3, HSA4 and HSA5 treatments, the flow behavior (Fig. 4d–f) after these post-treatments was completely different from that obtained after the HSA1 and HSA2 conditions, and manifested work hardening until failure. This behavior could be attributed to the combined effects of the presence of coarse carbide particles (brittle phase), which adversely affect the ductility, and the lower dislocation density in the initial microstructure of these treatment conditions prior to deformation (Fig. 5c,d). Thus, it can be concluded that the occurrence of the DRX process during the hot deformation of LPBF IN718 of the HSA1 and HSA2 conditions is attributable to the combined effects of the δ-phase along the grain boundaries and the presence of dislocation tangles inherited from the LPBF printing process.

**Figure 10:**
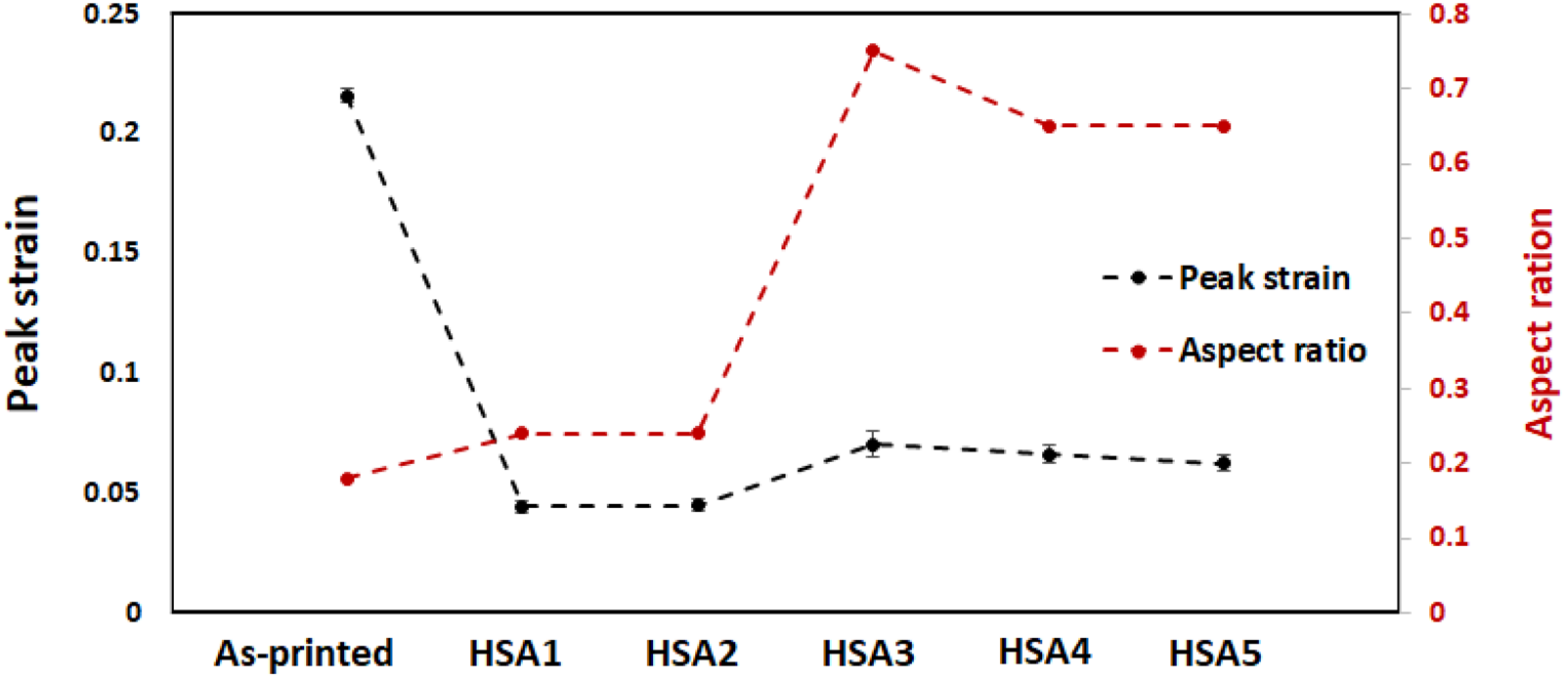
Evolution of the peak strain at 650 °C and aspect ratio with the heat treatment conditions.

To further understand this interaction, the evolution of the peak strain and the grain morphology represented by the grain aspect ratio as a function of the homogenization and solution treatment times was investigated, and is presented in Fig. 10. The aspect ratio reported in this figure corresponds to the grain width-to-length ratio, meaning that the closer this ratio to 1, the more equiaxed the grains are. The inclusion of the aspect ratio evolution in this figure is due to its importance to indicate the occurrence of the static recrystallization process during the heat treatments (prior to the hot deformation) that in turn reduce the primary dislocation density and refine the as-printed grain structure which are ones of the main driving forces of the DRX. As discussed above, the occurrence of the DRX can be indicated by the presence of a well-defined peak stress on the experimental true stress-true strain curve, as observed in Fig. 4b,c. However, the onset of the DRX process occurs at a specific strain value (critical strain, ε_c_) lower than that corresponding to the peak stress (peak strain, ε_p_). The value of the ${\upvarepsilon }_{c}$ is not easily extracted from the true stress-true strain curve, and some authors have used the relation of ${\upvarepsilon }_{c}= {a\upvarepsilon }_{\mathrm{p}}$ to identify the onset of the DRX, where *a* is a proportionality coefficient which ranges between 0.5 and 0.9^38^. Therefore, according to^38^, it is reasonable to assume that the peak strain is closely related to the critical strain for DRX since evaluating the ${\upvarepsilon }_{c}$ is out of focus of the present study.

Generally, after heat treatments, the peak strain significantly decreased, in contrast with the as-printed condition, as shown in Fig. 10. Several factors have been advanced in the literature to explain such a decrease in the critical/peak strain, including precipitations of secondary phases, decreases in the strain rate, decreases in the grain size prior to deformation, increases in the primary dislocation density and/or increases in the deformation temperature^38^. In the present study, the precipitation of secondary phases in the initial microstructure, such as δ, γ′ and γ′′, the retention of primary dislocation density and the refinement of as-printed grain structure in the initial microstructure, after some heat treatments, are more likely to be the driving force for the decrease in the peak strain after heat treatments since the hot deformation conditions (temperature and strain rate) did not change. Concerning the secondary phases, Yuan et al.^31^ found that the effect of the δ-phase on decreasing the peak strain is more significant than that of the γ′ and γ′′ phases. Among the post-treated conditions, both conditions of 1 h homogenization treatments (i.e., HSA1 and HSA2) exhibited the lowest peak strain, in contrast with the other treatments and the as-printed condition. Combined with the effect of the δ, γ′ and γ′′ phases, it can be confirmed that the retained dislocations network in the initial microstructure of the HSA1 and HSA2 conditions prior to deformation also contributed to the reduction in the peak strain.

Although the increase in the homogenization treatment time to 4 and 7 h in HSA3, HSA4 and HSA5 conditions resulted in a refinement of the as-printed microstructure by promoting complete recrystallization (static recrystallization), as indicated by the aspect ratio evolution, a relatively slight increase in the peak strain is observed, as compared to the HSA1 and HSA2 conditions. This can be explained by the annihilation of the initial dislocation network after these conditions, and confirms the significance of both the δ-phase and primary dislocation density in promoting the DRX during the hot deformation of IN718, and the lesser role of other driving forces such as grain refinement.

### Fracture surface analysis

**Figure 11:**
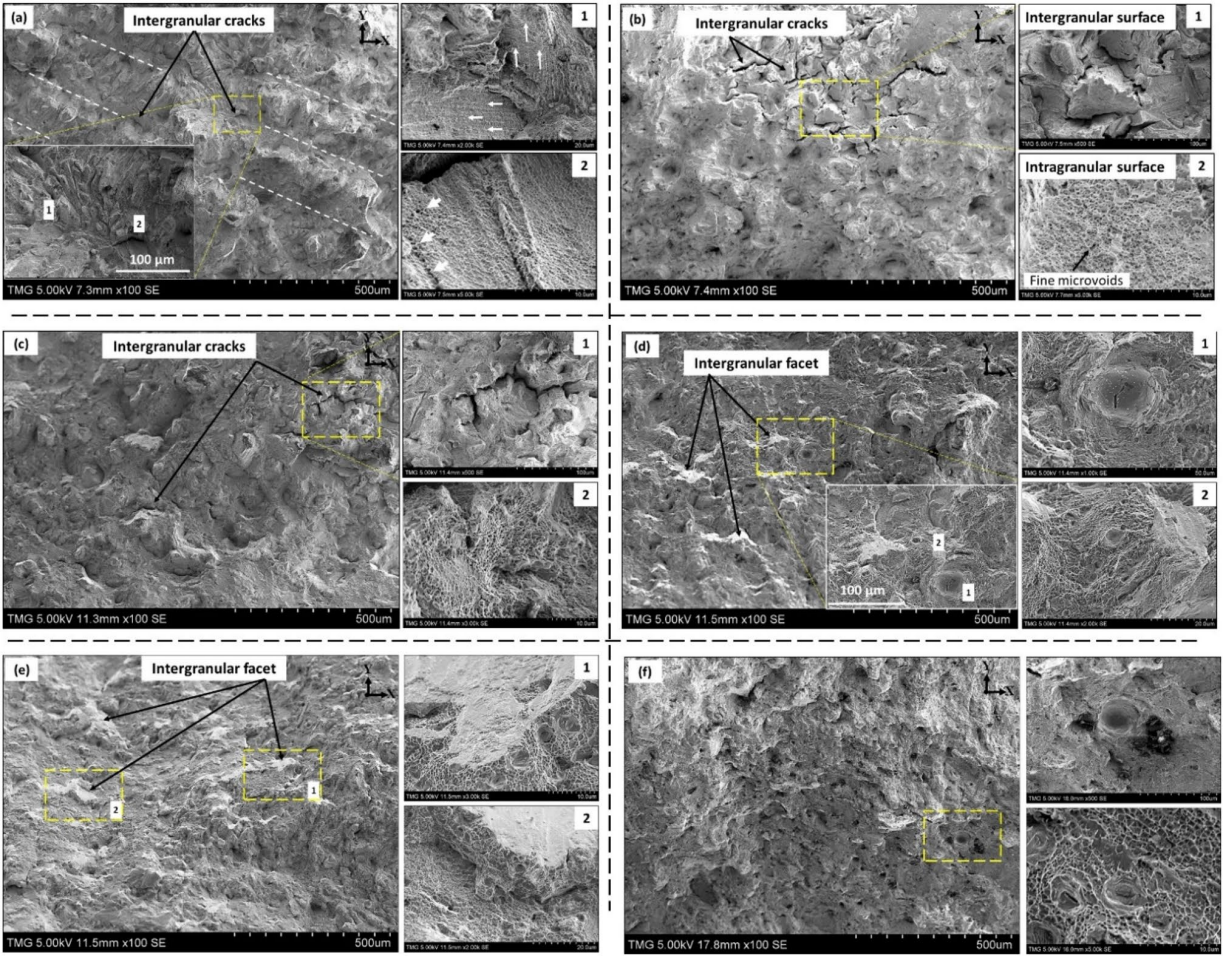
Fracture surface on the transverse plane of LPBF IN718: (**a**) as-printed; (**b**) HSA1; (**c**) HSA2; (**d**) HSA3; (**e**) HSA4; (**f**) HSA5. Fractures on melt-pool boundaries are highlighted by dotted lines in (**a**). Arrows in (a (1 and 2)) indicate the aligned intragranular dimples along the dendrites pattern in two different grains, and the coalescence of some microvoids along these dendrites, respectively.

The fracture surfaces of both the as-printed and heat-treated LPBF IN718 samples after tensile testing at 650 °C were investigated using an SEM to correlate the tensile characteristics with microstructures and determine the fracture mechanisms. Figure 11 illustrates the transverse cross-section (perpendicular to the tensile loading direction) of the tensile fracture surface of the as-printed and post-treated samples. In the as-printed condition, the cracks propagated in a mixed fracture mode: inter-dendritic (ductile), intergranular and inter-melt pool fractures. As can be seen in Fig. 11a, a pattern of parallel valleys-like shapes is observed, similar to the laser track pattern observed in the microstructure analysis of a transverse plane of the as-printed condition. The presence of this pattern indicates that large cracks propagated mainly along the melt pool boundaries. Such a fracture surface is consistent with that observed by Hilaire et al.^27^. Further clarification for these transverse cracks along the melt pool boundaries is shown in the facture surface of the longitudinal plane of the as-printed condition in the next section. In addition, large cracks are also observed along the grain boundaries, as shown in the inset image in Fig. 11a. The occurrence of the aforementioned intergranular and inter-melt pool fracture modes could be explained by the presence of relatively large brittle Laves precipitates along the melt pool boundaries, as well as the grain boundaries, which is consistent with the microstructural results^24^. It was confirmed in our previous work^24^ that the top border of the melt pool boundaries is occupied by relatively larger size Laves particles than those in the lower border because of the lower cooling rate in the top border. Crack nucleation and propagation thus occurs mainly along these borders. Furthermore, intragranular dimples aligned preferentially in a dendrite-like pattern and oriented differently from one grain to another were observed, as indicated by white arrows in Fig. 11a(1). Furthermore, some coalesced microvoids along the dendrite substructure were observed, and are indicated by white arrows in Fig. 11a(2). The presence of these microvoids is also attributable to the presence of Laves phase in the inter-dendritic regions.

On both HSA1 and HSA2 fracture surfaces, the inter-melt pool fracture mode (pattern of parallel valleys) and the preferred alignment of intragranular dimples disappeared completely. Such fracture surfaces are consistent with the microstructure analysis of the HSA1 and HSA2 conditions, where the as-printed features, such as melt pool boundaries, are eliminated. However, a mixture of intergranular and intragranular fracture modes was observed, as indicated by the presence of grain boundary cracks (see Fig. 11b(1),c(1)) and typical dimple feature (Fig. 11b(2),c(2)). Considering the microstructure analysis of both the HSA1 and HSA2 conditions, we see that the 1 h homogenization treatment at 1080 °C is not enough to completely dissolve Laves phase and cellular segregates, in addition to the precipitation of needle-like δ-phase along the grain boundaries^24^. Thus, large intergranular cracks and fine microvoids are observed in the areas occupied by the Laves and δ-phases, such as grain boundaries and inter-dendritic regions. In the HSA3, HSA4 and HSA5 conditions, the fracture surfaces show a mainly brittle mode, as indicated by the presence of mostly intergranular facets, as shown in Fig. 11d–f. The brittle fracture surface results from the embrittlement phenomenon discussed above and the presence of the coarse brittle MC carbides after HSA3, HSA4 and HSA5 treatments, which is confirmed by the microstructure and EDS analysis, shown in Fig. 8.

### Effect of Laves, carbides and δ-phase on the fracture behavior

**Figure 12:**
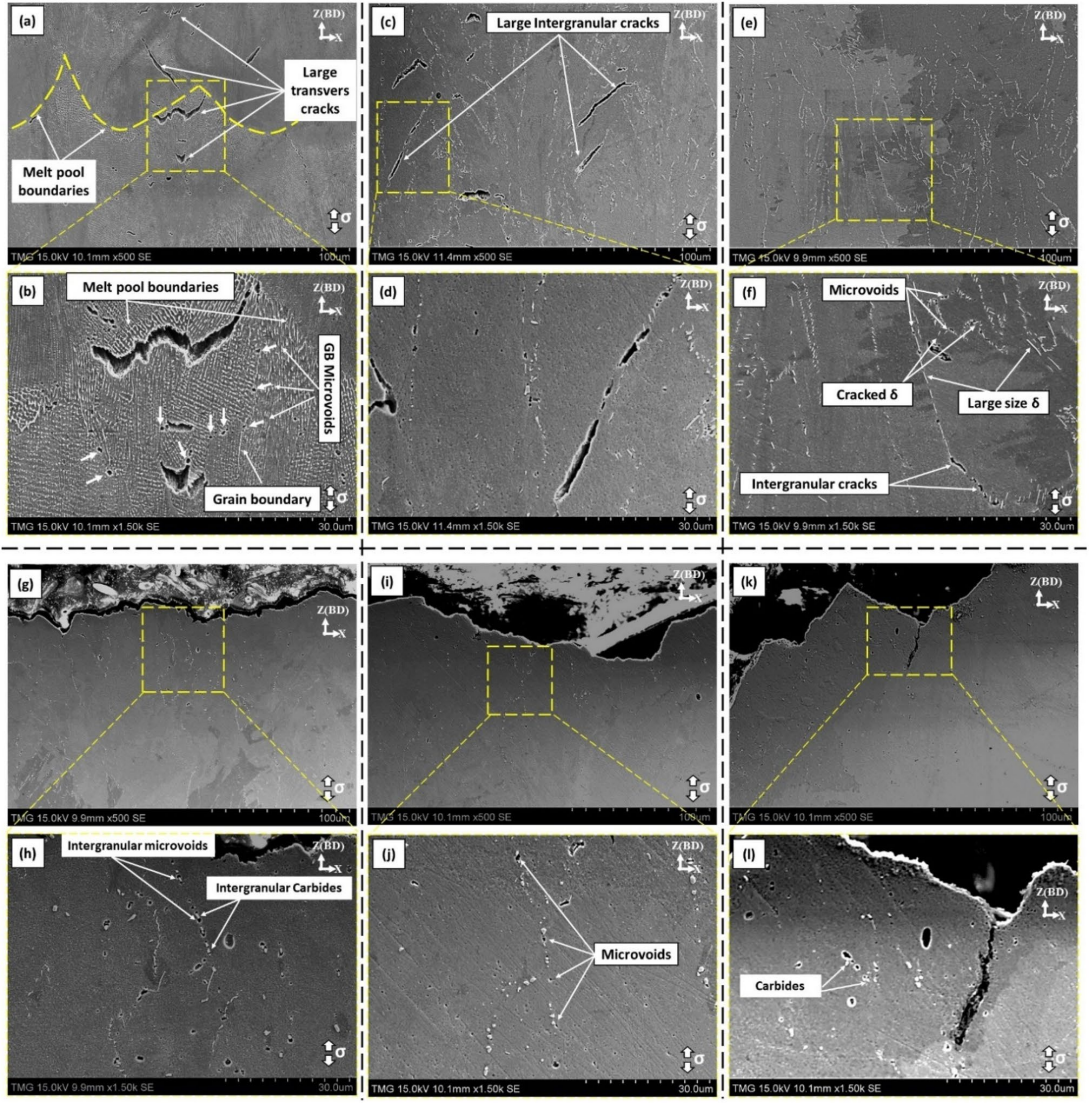
Fracture surface on the longitudinal plane of tensile testing of the LPBF IN718 at 650 °C: (**a**,**b**) as-printed; (**c**,**d**) HSA1; (**e**,**f**) HSA2; (**g**,**h**) HSA3; (**i**,**j**) HSA4; (**k**,**l**) HSA5. Arrows in (**b**) indicates the formation of microvoids along the grain boundaries and across the grains’ interior.

As discussed earlier, the heat treatment time window is established in a bid to optimize the heat treatment conditions for the LPBF IN718 by homogenizing the as-printed microstructure, dissolve the undesired phases, such as Laves phase, and control the precipitation of δ-phase to improve the mechanical behavior, especially at elevated temperatures. Thus, the effect of Laves, carbides and δ-phase on the fracture properties at 650 °C need to be examined. Figure 12 shows the longitudinal cross-section (parallel to the tensile loading direction) of the tensile fracture surface of the LPBF IN718 tensile samples in both the as-printed and post-treated conditions. As can be seen in Fig. 12a,b, in the as-printed condition, large transverse cracks (perpendicular to the building direction and tensile loading direction) are formed close to the melt pool boundaries. The presence of these transverse cracks could be explained by a high-stress level applied on the layer-layer melt pool boundaries, where a relatively large amount and size of brittle Laves phase formed. Furthermore, microvoids are observed along the grain boundaries and inter-dendritic zones, as indicated by white arrows in Fig. 12b. It is evident that Laves phases act as favorable sites for the formation of microvoids and crack propagation along the grain boundaries and melt pool boundaries.

In the HSA1 and HSA2 conditions, large intergranular cracks initiated and propagated along the grain boundaries where the Laves and δ-phases precipitated, as shown in Fig. 12c–f. It is worth noting that the δ-phase precipitating after HSA2 has a large size, and can easily crack and form voids, as shown in Fig. 12f. The dislocation accumulation near the δ-phase during the plastic deformation led to local stress concentration, causing microvoid and crack initiation near the δ-phase sites. By comparison, after the HSA3 treatment, the Laves phase almost completely dissolved, and a moderate amount of δ-phase precipitated, but more coarse MC carbides precipitated along the grain boundaries. Therefore, large voids are observed close to the coarse carbide particles, as shown in Fig. 12g,h. Similarly, after HSA4 and HSA5 treatments, large size voids formation is observed around the MC carbides. This is consistent with the significant ductility reduction in the IN718 after the application of HSA3, HSA4 and HSA5 treatments. Based on the known effect of Laves, δ and carbides on the fracture behavior, it is important to select the treatment having an intermediate homogenization time between HSA2 and HSA3 to completely dissolve the Laves phase, along with precipitation of smaller amounts and finer carbides in order to obtain a good combination of strength and ductility in the LPBF IN718 alloy. This condition could represent the starting point for further analysis in order to optimize the heat treatment conditions for LPBF IN718.

### Post-heat treatment map for LPBF IN718

**Figure 13:**
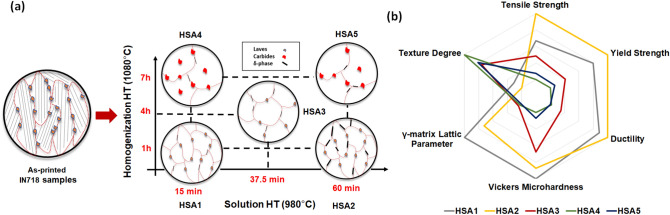
(**a**) Thermal post-processing map of LPBF IN718 alloy displaying the microstructure, phases and grain morphologies as functions of the heat treatment times; (**b**) radar chart summarizing the effect of the heat treatment time window on the texture, γ-matrix lattice parameter and mechanical properties.

Figure 13 shows the thermal post-heat treatment map of the LPBF IN718. The map was established using the information obtained in this and our previous study^24^ on the microstructure, precipitates, crystallographic texture, RT hardness and high temperature (650 °C) tensile properties of the LPBF IN718 alloy. It is established mainly to summarize and comprehensively understand the effect of the heat treatment time on the microstructural characteristics (phase transformation, homogeneity, grain structure and material texture) and mechanical properties of LPBF IN718 alloy at RT and 650 °C, and can be used as a decision making tool, when selecting the best-suited post-treatment conditions for LPBF IN718 alloy. It is worth mentioning that the texture degree, presented in Fig. 13b, is defined by the intensity ratio *I*_(111)_/*I*_(200)_, where the *I*_(111)_ and *I*_(200)_ designate the diffraction peak intensity of the planes of (111) and (200), respectively, which were obtained from the XRD analysis in the previous study^24^. Thus, the lowest values (fractions) represent a strong textured material, whereas values over unity refer to weak texture.

As can be seen in Fig. 13a, this map shows the development of the as-printed microstructure, which is characterized by directional solidification along the building direction, with fine particles precipitating along the grain boundaries and inter-dendritic regions. After thermal post-processing, a significant change in the as-printed microstructure, in terms of precipitates and grain structure, occurred, as illustrated in Fig. 13a. For the post-treated conditions, the map consists of two perpendicular axes: the vertical axis, illustrating the change in the microstructure, along with the homogenization treatment time at 1080 °C, and the horizontal axis, showing the microstructure change with the solution treatment time at 980 °C. Along the vertical axis, a significant change in the grain structure and texture is observed by increasing the duration of the homogenization treatment at 1080 °C: starting from a mixture of columnar and equiaxed grains at the relatively short treatment time (1 h), then on to more equiaxed recrystallized grains at the intermediate soaking time (4 h), and finally, coarse equiaxed grains after the prolonged treatment condition (7 h). Furthermore, more Lave phase dissolution is observed by increasing the homogenization treatment duration. However, the intermediate (4 h) and the prolonged (7 h) homogenization treatment times resulted in greater precipitations of coarse MC carbides. Moreover, it was found that the homogenization treatment time has a significant impact on the mechanical properties at RT and 650 °C. The 1 h homogenized conditions exhibited the highest RT hardness and tensile properties at 650 °C due to the effect of dislocation tangles and the relatively higher contents of the Nb and Ti released into the γ-matrix, as indicated by the calculated lattice parameter in the radar chart in Fig. 13b, which are necessary for precipitation of γ′ and γ′′ as compared to the longer homogenization time.

Along the horizontal axis, the solution heat treatment time at 980 °C does not impose a noticeable change in the grain structure. However, the amount of precipitated δ-phase was significantly affected by the solution treatment time. Increasing the duration of the solution treatment resulted in an increase in δ-phase amount. Also, the precipitation of δ-phase along the grain boundaries plays an important role in improving the tensile strength of LPBF IN718 alloys at 650 °C. Thus, the HSA2 condition exhibited a relatively higher strength than that of the HSA1 condition, as shown in Fig. 13b, due to the increased precipitation of δ-phase at the grain boundaries in the former condition. The same behavior is observed between the HAS4 and HSA5 conditions. Based on the observations and analysis of the results obtained so far, there is a conflict between the mechanical properties and the material texture since the highest mechanical properties are obtained after applying the conditions (HAS1 and HSA2) under which the as-printed strong texture does not change significantly. Thus, an intermediate homogenization holding time of between two and four hours at 1080 °C could help produce IN718 parts with high and isotropic mechanical properties.

## Conclusion

In the present study, the influence of the homogenization and solution treatment times on the elevated-temperature (650 °C) mechanical properties of LPBF-fabricated IN718 has been investigated. The main findings can be summarized as follows:

- The as-printed IN718 shows the lowest strength, but the highest ductility, as compared to the post-heat-treated conditions, due to a combined effect of the absence of γ′ and γ′′ phases and the precipitation of Laves phase. Moreover, high elongations in the as-printed conditions are attributable to the as-printed grain structure, with grains elongated along the tensile loading direction. The Laves phase in the as-printed IN718 provides a favorable site for microvoids nucleation and crack propagation.
- After heat treatments, the tensile and yield strength significantly increased by 20.3–31% and 46.1–62.3%, respectively, as compared to the as-printed condition, depending on the solution and homogenization treatment time, while the ductility correspondingly decreased by 67.4–80%.
- Among the heat-treated conditions, the highest strength and ductility were obtained after the 1 h homogenization treatment conditions, HSA1 and HSA2, due to the relatively high content of γ′ and γ′′ phases and dislocation density, as compared to other heat treatments.
- Further increases in the homogenization treatment time in the HSA3, HSA4 and HSA5 conditions resulted in a significant loss of ductility of LPBF IN718 at 650 °C. This is attributable to the increase in the precipitation of coarse MC carbide particles along the grain boundaries after these conditions.
- The presence of δ-phase at the grain boundaries improved the elevated-temperature mechanical properties of IN718 alloy. Thus, the HSA2 condition resulted in higher strength than did the HSA1 condition, which was related to an increased precipitation of δ-phase after HSA2.
- The intergranular δ-phase accelerates the dynamic recrystallization process during the hot deformation of IN718 alloy. Furthermore, dynamic recrystallization process is promoted only after the application of HSA1 and HSA2 due to a combined effect of the initial dislocation network, as well as the presence of δ-phase along the grain boundaries.
